# Molecular determinants of nucleic acid recognition by an RNA-targeting ADP-ribosyltransferase toxin

**DOI:** 10.1016/j.jbc.2025.110463

**Published:** 2025-07-07

**Authors:** Fanyang Lv, Andrea G. Alexei, Jake Colautti, Nathan P. Bullen, John C. Whitney

**Affiliations:** 1 Michael DeGroote Institute for Infectious Disease Research, McMaster University, Hamilton, Ontario, Canada; 2 Department of Biochemistry and Biomedical Sciences, McMaster University, Hamilton, Ontario, Canada; 3 David Braley Centre for Antibiotic Discovery, McMaster University, Hamilton, Ontario, Canada

**Keywords:** ADP-ribosylation, bacterial toxin, RNA, RNA-binding protein, RNA modification

## Abstract

ADP-ribosyltransferases use NAD^+^ to catalyze ADP-ribosylation reactions that regulate diverse cellular pathways in eukaryotes or function as toxins delivered by bacteria to kill competitor or host cells. Although most characterized ARTs target proteins, we recently identified RhsP2 as an antibacterial ART toxin that modifies the 2′-OH groups of structured RNAs during bacterial competition. However, the molecular basis for RhsP2’s unique specificity toward RNA remains poorly understood. Here, we show that RhsP2 is a divergent member of the ART superfamily that recognizes nucleic acid substrates *via* a positively charged RNA-binding surface adjacent to its catalytic site. Mutations within this surface disrupt both RNA binding and ADP-ribosylation activity, abolishing RhsP2’s antibacterial function. We further demonstrate that RhsP2 binds distinct small regulatory RNAs with varying affinities, suggesting that both electrostatic interactions and shape complementarity contribute to RNA target selection. Together, our findings define the molecular determinants of nucleic acid recognition by an unusual RNA-targeting ART toxin.

ADP-ribosyltransferases (ARTs) are a ubiquitous family of enzymes expressed in organisms from all three phylogenetic domains that catalyze the transfer of ADP-ribose (ADPr) from nicotinamide adenine dinucleotide (NAD^+^) onto target macromolecules (1, 2). This modification, known as ADP-ribosylation, plays a central role in regulating diverse cellular processes, including DNA repair, transcription, signal transduction, and metabolism (3, 4, 5, 6). In addition to these regulatory functions, several bacterial ARTs act as toxins that disrupt cellular physiology, contributing to bacterial virulence, interbacterial competition, and defense against bacteriophage infection (1, 5, 7, 8, 9). ARTs exhibit broad substrate specificity despite sharing a conserved catalytic mechanism, which likely underlies their participation in a wide range of biological processes (1, 7, 9).

In eukaryotes, ART substrate specificity is often governed by distinct substrate-recognition domains encoded within the same polypeptide as the catalytic ART domain (10, 11). This arrangement is exemplified by the poly (ADP-ribose) polymerase (PARP) family, which comprises multiple ARTs that use diverse targeting domains to recognize their cognate substrates with high specificity. For instance, PARP1 contains two zinc finger domains that detect DNA damage, enabling it to ADP-ribosylate histones at damage sites and recruit DNA repair factors (8, 12, 13, 14, 15). PARP5A and PARP5B, on the other hand, harbor ankyrin-repeat domains that bind defined peptide motifs within target proteins (16, 17).

Unlike their eukaryotic counterparts, bacterial ARTs typically lack auxiliary substrate-recognition domains. Instead, for these enzymes, substrate specificity is encoded within the catalytic ART domain itself through structural motifs that mediate direct interactions with the modification target. A well-characterized example is the cholera toxin (CTX) family of ARTs, in which a structural feature known as the ADP-ribosylating turn-turn (ARTT) loop binds protein substrates and positions them within the active site for modification (7, 8, 18, 19, 20). Members of the diphtheria toxin (DTX) family lack the ARTT loop and instead employ alternative substrate recognition strategies (5, 7, 21, 22). For instance, *Pseudomonas* exotoxin A recognizes the unique diphthamide-modified residue of eukaryotic elongation factor 2 (eEF-2) *via* its active-site loops, whereas another member of this family, cholix toxin, uses a distinct region known as Loop 1 to target the same residue (23, 24, 25). Collectively, these examples highlight how bacterial ARTs have evolved built-in recognition features within their core catalytic fold to achieve precise substrate targeting that obviate the need for additional specificity domains.

Although ARTs were once thought to exclusively modify proteins, recent studies demonstrate that some bacterial ARTs instead target nucleic acids (26). The best-characterized example is the toxin component of the DarTG toxin–antitoxin system (DarT), which modifies single-stranded DNA by ADP-ribosylating thymidine residues within specific sequence motifs (27). DNA modification by DarT plays a role in regulating bacterial growth or serves as a defense mechanism against phage infection (27, 28). Structural studies showed that DarT DNA-targeting activity is facilitated by a positively charged surface near the enzyme’s active site that positions the substrate for catalysis by interacting with the DNA backbone (29). These findings suggest that like protein-targeting ARTs, nucleic acid-targeting ARTs rely on features within their catalytic domains to achieve substrate specificity. However, aside from DarT, the molecular basis for nucleic acid recognition by ARTs remains largely unexplored. Furthermore, because DarT is strictly DNA-specific, it is unclear whether other nucleic acid-targeting ARTs recognize their substrates through similar mechanisms.

In recent work, we identified RhsP2 as an RNA-targeting ART that functions as an antibacterial toxin secreted by the H2 type VI secretion system (H2-T6SS) of *Pseudomonas aeruginosa* (30). Our initial characterization revealed that RhsP2 specifically catalyzes ADP-ribosylation of structured RNA molecules, including all tested tRNAs and the RNA subunit of the tRNA-processing enzyme ribonuclease P (RNase P). These modifications disrupt polycistronic tRNA processing and inhibit translation, ultimately leading to bacterial growth arrest (30). However, the molecular basis for RNA substrate recognition by RhsP2 remains unknown.

In this study, we investigate the structural and biochemical features of RhsP2 that enable its RNA-targeting activity. Through a combination of biochemical assays, structural modeling, and interbacterial competition experiments, we identify a positively charged surface adjacent to the RhsP2 active site that mediates RNA binding. Within this surface, we pinpoint lysine 1574 (K1574) as the residue most essential for RNA substrate binding. Substitution of K1574 with glutamate abolishes RNA binding and ADP-ribosylation activity whereas substitution of other nearby basic residues reduces RhsP2’s affinity for RNA. Together, these findings uncover the molecular basis for RNA recognition by RhsP2 and broaden our understanding of substrate targeting mechanisms employed by nucleic acid-modifying ART toxins.

## Results

### RhsP2 possesses a non-canonical active site configuration

**Figure 1:**
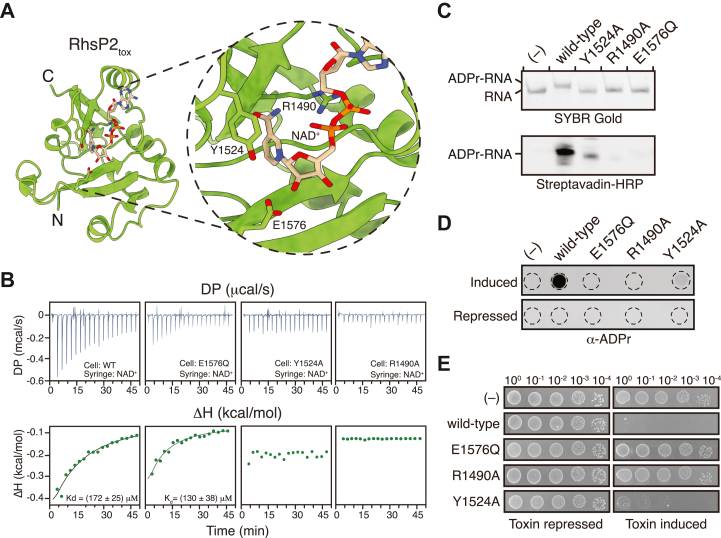
**Structural and functional characterization of RhsP2_tox_ as an RNA-targeting ART.***A*, alphaFold3-predicted NAD^+^ binding site using the X-ray crystal structure of RhsP2_tox_ (chain A from PDB code: 7RT7). Key residues involved in NAD^+^ binding and catalysis are shown as sticks. *B*, NAD^+^ binding to wild-type and mutant RhsP2_tox_ protein measured by ITC. Raw titration curves (*top*) and fitted Wiseman isotherms (*bottom*) are shown. Binding affinities were calculated using a one-binding-site model. *C*, *in vitro* ADP-ribosylation activity of wild-type RhsP2_tox_ and the indicated NAD ^+^ -binding and catalytic site mutants. Reactions were performed using biotin-NAD^+^ as the substrate, and products were analyzed by SYBR Gold staining (*top*) and western blotting with streptavidin–HRP (*bottom*). *D*, dot blot analysis of total cellular RNA using an anti-ADP ribose antibody. RNA was isolated from cells expressing wild-type RhsP2_tox_ or the indicated mutants and spotted onto cationic nylon membranes to detect ADP-ribosylation *via* dot blot. *E*, toxicity assay examining the growth of *E. coli* XL-1 Blue under toxin-repressed and toxin-induced conditions. The assay compares the effects of wild-type RhsP2_tox_ and the indicated NAD^+^ binding or catalysis mutants on bacterial growth. In panels *B*, *D*, and *E*, “(−)” indicates a no toxin condition (*B*) or an empty vector control (*D* and *E*).

ART toxins are traditionally classified as either DTX or CTX based on the conserved catalytic motifs H-Y-E or R-S-E, respectively (5, 7, 31). In our initial characterization of RhsP2, we found that it uniquely combines elements of both motifs, containing an R-Y-E catalytic triad (30). Using the recently released diffusion module of AlphaFold3, we predicted the NAD^+^-binding mode within the RhsP2 active site and found that all three conserved residues participate in substrate engagement similar to what is observed for DTX and CTX ARTs (Figs. 1*A* and S1, *A* and *B*). Like cholera toxin-like ARTs, R1490 interacts electrostatically with the pyrophosphate group of NAD^+^ (32). Additionally, Y1524 is positioned similar to a key tyrosine in DTX-like toxins that contacts the nicotinamide moiety of NAD^+^ through π-π stacking (33, 34). E1576 is a highly conserved catalytic glutamate located at the beginning of the β-5 strand that is found in almost all of ART enzymes and is thought to stabilize the oxocarbenium ion transition state in order to provide catalysis (4, 5, 35, 36). Altogether, our analysis suggests that despite the novel catalytic triad of RhsP2, the residues are likely to act in a similar manner to that of other ARTs to mediate enzymatic activity.

To assess the functional contributions of the conserved R-Y-E residues in RhsP2, we performed isothermal titration calorimetry (ITC) on site-specific variants of the C-terminal ART domain (RhsP2_tox_). These experiments revealed that mutations in either R1490 or Y1524, but not E1576, impaired NAD^+^ binding, consistent with their predicted roles in substrate interaction and catalysis, respectively (Fig. 1*B*). We next assessed enzymatic activity using a previously described 50-nt synthetic RNA oligonucleotide (30). *In vitro* ADP-ribosylation assays confirmed that RhsP2_tox_ variants R1490A and E1576Q are catalytically inactive whereas Y1524A exhibits markedly reduced activity (Fig. 1*C*). In line with these biochemical findings, expression of the same variants in *Escherichia coli* revealed no detectable ADP-ribosylated RNA for R1490A and E1576Q, and only low levels for Y1524A (Fig. 1*D*). Interestingly, despite its reduced enzymatic activity, the Y1524A variant retains bactericidal activity in *E. coli*, consistent with previous growth inhibition studies (Fig. 1*E*) (30). Taken together, these results indicate that the conserved R-Y-E residues in RhsP2 fulfill roles analogous to those of canonical ART toxins, although RhsP2 uniquely targets RNA substrates.

### RNA binding by RhsP2 is mediated by positively charged residues adjacent to the active site

**Figure 2:**
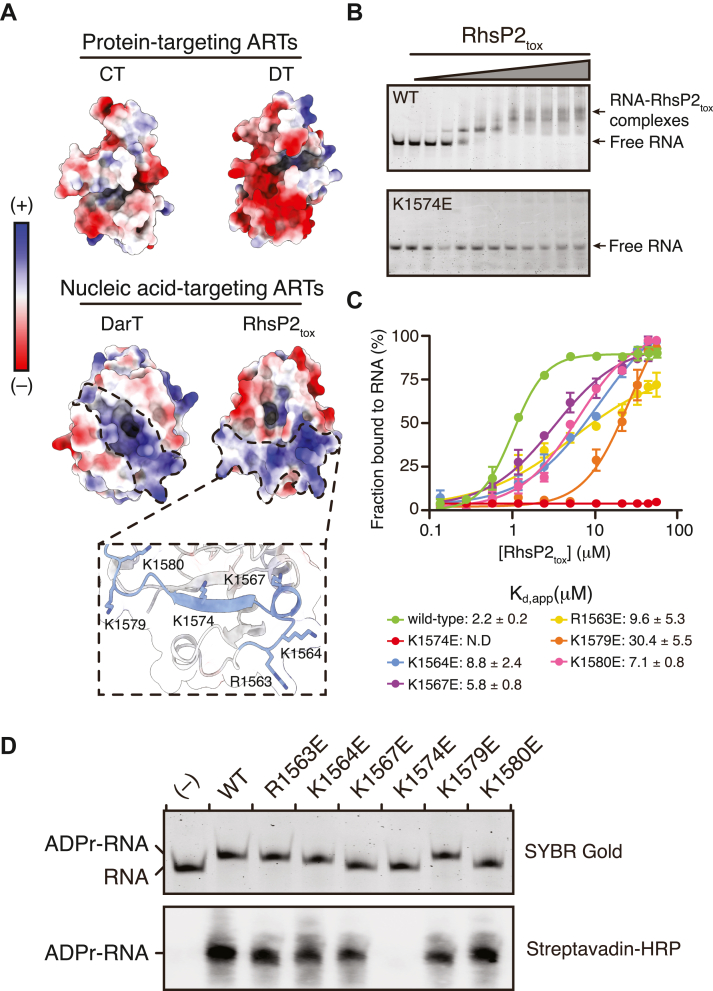
**RhsP2_tox_ utilizes electrostatic interactions for RNA substrate recognition.***A*, electrostatic surface potential comparison of representative ART family members, including CT (cholera toxin, PDB: 2A5G), DT (Diphtheria toxin, PDB, 1DTP), DarT (PDB, 70MV), RhsP2_tox_ (PDB, 7RT7). For DarT and RhsP2_tox_, positively charged regions near the catalytic site are highlighted. Inset shows a zoom in of RhsP2_tox_’s positively charged surface shown in cartoon format, with putative RNA-binding residues highlighted in *blue*. *B*, RNA-binding activity of RhsP2_tox_ and its point mutants analyzed by electrophoretic mobility shift assays (EMSA). Representative gel images showing the binding of a 50-nt RNA to RhsP2_tox_ and its K1574 mutant. Complexes were separated on a native polyacrylamide gel and visualized by SYBR Gold staining. *C*, binding curves for wild-type RhsP2_tox_ and its mutants, showing relative RNA-binding affinities. Apparent dissociation constants (*K*_d,app_) obtained from nonlinear regression using the specific binding model with a Hill slope are indicated, and representative gel images can be found in Fig. S2. N.D., not detected. *D*, *in vitro* ADP-ribosylation activity of wild-type RhsP2_tox_ and the indicated RNA-binding mutants. Reactions were performed using biotin-NAD^+^ as the substrate and products were analyzed by either SYBR Gold staining (*top*) or western blotting with streptavidin-HRP (*bottom*).

Having elucidated the molecular contacts involved in NAD^+^ binding by RhsP2, we next sought to determine how the toxin recognizes RNA substrates. Canonical RNA-binding proteins often contain defined RNA-binding domains such as the ribonucleoprotein (RNP) motif, zinc finger domains, or K homology domains (37, 38, 39). However, sequence and structural analyses revealed that the 1614-residue RhsP2 protein lacks homology to any known RNA-binding domains and thus its RNA-binding capability is intrinsic to the ART domain itself. To investigate this possibility, we compared the surface properties of RhsP2_tox_ with those of several well-characterized ARTs that modify either proteins or nucleic acids. This analysis revealed a prominent positively charged surface adjacent to RhsP2’s active site—a feature that is absent in protein-targeting ARTs but resembles the electropositive substrate-binding groove found in the DNA-targeting DarT toxin (Fig. 2*A*) (29). Drawing on this similarity, we hypothesized that RhsP2 binds RNA substrates through a similar charge-based interaction. Examination of our previously determined RhsP2_tox_ crystal structure identified six basic residues—R1563, K1564, K1567, K1574, K1579, and K1580—that collectively define this electropositive surface (Fig. 2*A*).

To investigate the contribution of these basic residues to RNA binding, we performed electrophoretic mobility shift assays (EMSAs) using wild-type and mutant RhsP2_tox_ proteins. RhsP2’s catalytic activity depends on the presence of NAD^+^. Therefore, in the absence of NAD^+^, RhsP2_tox_ is expected to bind RNA without modifying it, allowing us to assess substrate-binding affinity independent of catalysis. Additionally, because EMSAs do not measure equilibrium binding, we report all experimentally derived binding affinities as apparent dissociation constants (*K*_d,app_). Using the aforementioned synthetic 50-nt RNA oligonucleotide as a starting point, we found that wild-type RhsP2_tox_ binds to this RNA with micromolar affinity (*K*_d,app_ ≈ 2.2 μM; Fig. S2, *B* and *C*). Charge reversal mutations at each of the candidate RNA-binding residues reduced RNA binding to varying degrees, with the K1574E variant showing a complete loss of detectable RNA binding (Figs. 2, *B* and *C* and S2). To assess the functional consequences of these binding defects, we measured the ADP-ribosylation activity of each RhsP2_tox_ variant using the same 50-nt RNA substrate in the presence of NAD^+^. Consistent with the EMSA results, the K1574E mutation abolished catalytic activity as determined by both *in vitro* RNA ADP-ribosylation assays and biotin-NAD^+^ labeling (Fig. 2*D*). Overall, these findings support a model in which RhsP2 recognizes RNA substrates *via* a positively charged surface adjacent to the active site, with K1574 playing a central role in engaging the RNA backbone and facilitating substrate positioning for catalysis.

### RhsP2 modifies biologically relevant sRNAs *via* a conserved RNA-binding interface

To determine whether the positively charged surface of RhsP2 mediates binding to biologically relevant RNA targets, we selected two small regulatory RNAs (sRNAs) for further study: CsrB and McaS. CsrB was previously shown to be modified by RhsP2 in our earlier study, establishing its relevance as a substrate (30). McaS, on the other hand, is a well-characterized sRNA involved in regulating gene expression and biofilm formation that has not been previously examined as a potential substrate of RhsP2 (40, 41). Both RNAs are highly structured and can be efficiently synthesized in large quantities by *in vitro* transcription, making them ideally suited for binding and modification studies (Fig. S3).

**Figure 3:**
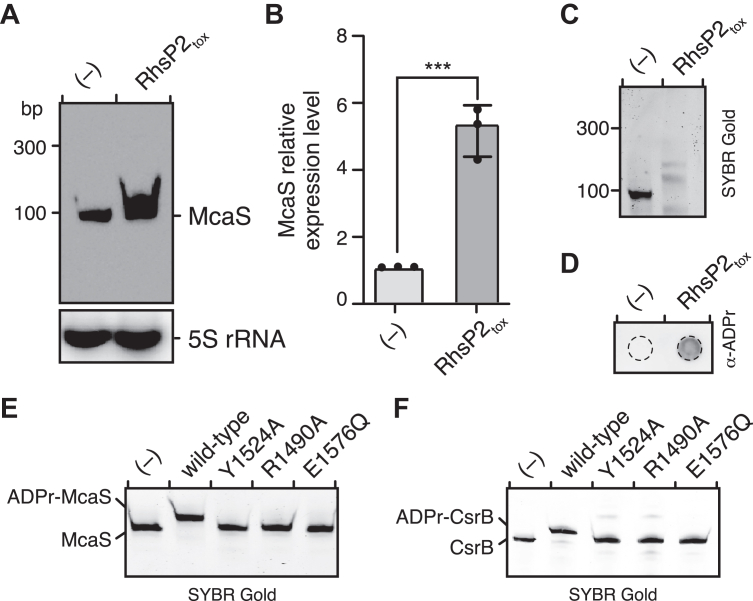
**RhsP2 ADP-ribosylates the sRNAs McaS and CsrB.***A*, northern blotting analysis of McaS from cells expressing RhsP2_tox_ or an empty vector control (−) after 30 min post-induction. 5S rRNA levels serve as a loading control. *B*, quantification of McaS relative expression level in cells expressing either an empty vector (−) or RhsP2_tox_, normalized to 16S rRNA levels. *C*, SYBR Gold stained gels showing the purified McaS from RNA pull-down assays. *D*, ADP-ribosylation dot blot of the purified McaS samples from (*C*), detected using an anti-ADP ribose antibody. *E* and *F*, *in vitro* ADP-ribosylation activity by wild-type RhsP2_tox_ and the indicated catalytic site mutants of McaS (*E*) and CsrB (*F*). “(−)” indicates a vector control strain (*A*–*D*) or a toxin-free reaction condition (*E* and *F*).

To first assess whether McaS is a substrate of RhsP2, we performed northern blot analysis on total RNA extracted from *E. coli* under toxin-induced and toxin-repressed conditions. Upon RhsP2_tox_ expression we observed a mobility shift of the McaS transcript that is consistent with the covalent addition of one or more ADP-ribose moieties to this RNA (Fig. 3*A*). Quantitative RT-PCR further revealed increased levels of McaS RNA in the presence of RhsP2_tox_, potentially reflecting stabilization of the modified RNA or an indirect transcriptional response (Fig. 3*B*). Pull-down assays using a biotin-labeled DNA probe specific to McaS followed by immunoblotting with an ADP-ribose specific antibody confirmed that McaS is ADP-ribosylated *in vivo*, establishing it as a direct substrate of RhsP2 (Fig. 3, *C* and *D*).

**Figure 4:**
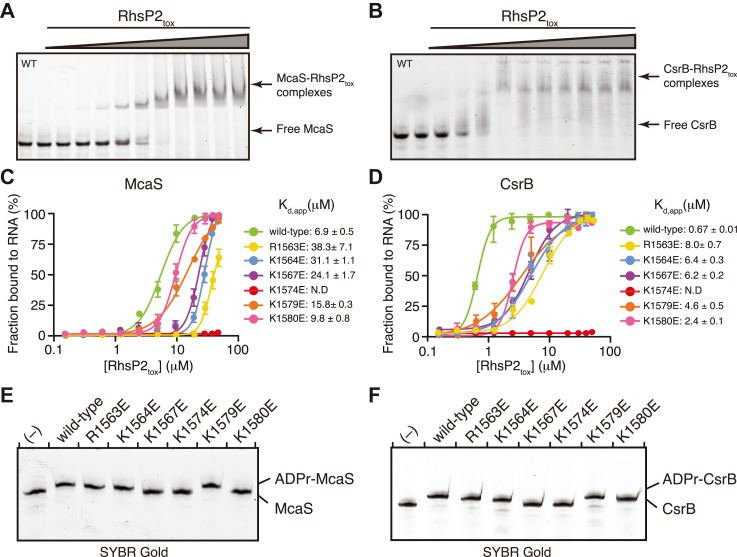
**RNA-binding and ADP-ribosylation activities of RhsP2_tox_ towards McaS and CsrB**. *A* and *B*, RNA-binding activity of RhsP2_tox_ to McaS (*A*) and CsrB (*B*) analyzed by native electrophoretic mobility shift assays (EMSA). Assays were performed in duplicate and representative gel images are shown. Gels were visualized by SYBR Gold staining. *C* and *D*, equilibrium binding curves for wild-type RhsP2_tox_ and the indicated mutants for McaS (*C*) and CsrB (*D*) showing RNA-binding affinities. Apparent dissociation constants (K_d,app_) for each toxin-RNA complex are indicated (n = 2). N.D, not detected. *E* and *F*, *in vitro* ADP-ribosylation activity of RhsP2_tox_ and the indicated RNA-binding mutants using McaS (*E*) and CsrB (*F*) as substrates. Reaction products were visualized after 30 min incubation. “(−)” indicates a toxin-free reaction condition.

We next investigated whether the positively charged RNA-binding surface of RhsP2 is required for recognition of these structured sRNAs. EMSAs using *in vitro* transcribed McaS and CsrB revealed that wild-type RhsP2_tox_ binds both RNAs with very different apparent affinities (*K*_d,app_ ≈ 6.9 μM for McaS and 0.67 μM for CsrB) (Fig. 4, *A*–*D*). By contrast, charge-reversal mutants targeting residues within the RNA-binding interface—including R1563E, K1564E, K1567E, K1574E, K1579E, and K1580E—showed significantly reduced RNA-binding capacity (Figs. 4, *C* and *D* and S4). Notably, the K1574E mutation completely abolished detectable binding to both sRNAs, consistent with results obtained using the synthetic 50-nt RNA substrate. These findings support the conclusion that K1574 is a key determinant of RNA binding for a range of structured RNAs.

To determine whether these binding defects also impair enzymatic activity, we assessed RNA ADP-ribosylation by wild-type and mutant RhsP2_tox_ proteins in the presence of NAD^+^. As expected, wild-type RhsP2_tox_ efficiently ADP-ribosylated both McaS and CsrB as measured by *in vitro* modification assays (Fig. 4, *E* and *F*). By contrast, the catalytically impaired mutants (R1490A, Y1524A, and E1576Q) and the RNA-binding surface mutants K1567E and K1574E failed to modify either RNA substrate (Figs. 3, *E* and *F* and 4, *E* and *F*). Interestingly, some RNA-binding mutants exhibited substrate-specific effects, with partial modification observed for one RNA but not the other (*e.g.* K1580), suggesting that individual residues within the electropositive interface may contribute to differential recognition of distinct sRNAs.

Taken together, these results demonstrate that RhsP2_tox_ recognizes and modifies biologically relevant structured RNAs using a conserved cationic surface centered around K1574. Although this interface is broadly permissive and supports binding to multiple RNA substrates, subtle differences in affinity and modification efficiency suggest that electrostatic complementarity is modulated by structural features unique to each RNA target.

### The RNA-binding interface of RhsP2 is required for its antibacterial activity

**Figure 5:**
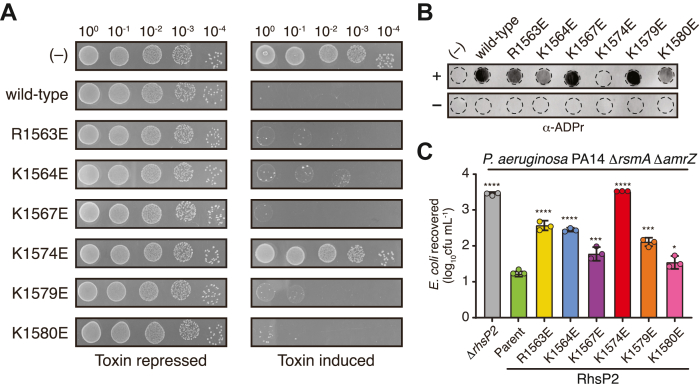
**Functional importance of RhsP2tox RNA-binding sites *in vivo*.***A*, toxicity assay monitoring the growth of *E. coli* XL-blue under uninduced and induced conditions of wild type RhsP2_tox_ and its RNA-binding site mutants. *B*, *dot blot* analysis of total RNA samples using an anti-ADP ribose antibody. RNA isolated from cells expressing wild-type RhsP2_tox_ and RNA-binding mutants was spotted onto positively charged nylon membranes to detect ADP-ribosylation. *C*, outcome of growth competition assays between an *E. coli* recipient and the indicated *P. aeruginosa* PA14 donor strains. All mutants are derived from the parental strain (Δ*rsmA*Δ*amrZ*) background. The data represent means ± SD from three biological replicates. *Asterisks* indicate statistical significance, determined using one-way ANOVA followed by Tukey’s *post hoc* test, in recovered *E. coli* compared with the parental donor strain (∗∗∗∗*p* ≤ 0.0001, ∗∗*p* ≤ 0.001, ∗*p* ≤ 0.05). “(−)” indicates a vector control (*A*) or non-toxin-expressing strain (*B*).

Having established that a cationic surface adjacent to RhsP2’s active site mediates RNA binding and ADP-ribosylation *in vitro*, we next investigated whether this surface is required for RhsP2-mediated toxicity *in vivo*. Using a heterologous expression system in *E. coli*, we found that charge reversal mutations within this surface impaired the growth inhibitory properties of the toxin, consistent with their compromised RNA-binding and catalytic activity (Fig. 5*A*). Notably, the substitution of the central K1574 residue to glutamate (K1574E) abolished toxicity, mirroring its loss of RNA-binding and enzymatic function in biochemical assays.

To test whether the observed growth inhibition phenotypes result from impaired RNA ADP-ribosylation *in vivo*, we assessed RNA ADP-ribosylation in *E. coli* cells expressing wild-type or each of the mutant RhsP2_tox_ proteins. Total RNA was extracted from toxin-induced and uninduced cultures and analyzed by dot blot using an anti-ADP-ribose antibody. Robust RNA modification was observed in cells expressing wild-type toxin whereas the K1574E variant showed no detectable ADP-ribosylation, consistent with its loss of RNA-binding and enzymatic activity *in vitro*. By contrast, other RNA-binding interface mutants (R1563E, K1564E, K1567E, K1579E, and K1580E) displayed intermediate levels of RNA ADP-ribosylation, in line with their partial reduction in RNA binding affinity observed in EMSA assays (Fig. 5*B*). These results further support the conclusion that the cationic RNA-binding surface, particularly K1574, is required for efficient ADP-ribosylation *in vivo*.

To test whether this surface is required for toxicity during T6SS-dependent bacterial competition, we engineered chromosomal point mutations of the RNA-binding surface into *P. aeruginosa* PA14 and evaluated their impact on the ability of *P. aeruginosa* to outcompete *E. coli* in an RhsP2-dependent manner. Because the T6SS of *P. aeruginosa* that delivers RhsP2 into competitor bacteria (H2-T6SS) is minimally expressed under standard laboratory conditions, we used a previously described Δ*rsmA* Δ*amrZ* genetic background to increase its expression levels (42). Using this parent strain, *P. aeruginosa* exhibits a strong H2-T6SS- and *rhsP2*-dependent co-culture fitness advantage over *E. coli*. However, strains harboring charge reversal mutations in RhsP2’s RNA-binding interface showed reduced interbacterial antagonism. As was observed for our *E. coli* expression system, the K1574E mutant displayed a near-complete loss of RhsP2-dependent *E. coli* killing, comparable to that of a Δ*rhsP2* control strain (Fig. 5*C*). Taken together, these findings demonstrate that the cationic RNA-binding surface of RhsP2, and K1574 is essential for the protein’s ability to bind and modify RNA *in vivo* and is required for its function as a T6SS-delivered antibacterial toxin.

## Discussion

In this study, we define the molecular mechanism by which the antibacterial toxin RhsP2 recognizes RNA substrates. Building on our prior work that established RhsP2 as an RNA-targeting ADP-ribosyltransferase (ART) toxin, we now identify the structural determinants that underlie its RNA-binding properties (30). Specifically, we show that RhsP2 employs a conserved, positively charged surface adjacent to its active site to bind RNA substrates. Mutations in this surface, particularly at K1574, impair both RNA binding and ADP-ribosylation activity, abolishing the toxin’s antibacterial function *in vitro* and during interbacterial competition. These findings reveal a predominantly electrostatics-based mechanism of RNA recognition and thus expand our understanding of nucleic acid-targeting enzymes within the ART superfamily.

While the majority of characterized ARTs modify proteins, a growing number of studies demonstrate that nucleic acids also serve as substrates. The best-understood example is DarT, a toxin that modifies single-stranded DNA by ADP-ribosylating thymidine residues within specific sequence motifs (27, 29). DarT relies on an electropositive groove near its active site to engage the DNA backbone, a strategy we now show is recapitulated by RhsP2 in its recognition of RNA. Our structural and mutational analyses reveal that RhsP2 harbors a similarly positioned cationic interface composed of six basic residues. This surface lacks any sequence or structural similarity to canonical RNA-binding domains and thus appears to represent an adaptation for targeting the negatively charged RNA backbone that is functionally analogous to DarT. Of the residues that make up RhsP2’s cationic surface, K1574 is uniquely essential in that mutation of this site abolishes RNA binding, enzymatic activity, and toxicity, both *in vitro* and *in vivo*.

Although RhsP2 and DarT share a similar electropositive substrate-binding groove, a key distinction lies in the relative positioning of their NAD^+^-binding pocket. In DarT, the NAD^+^ binding site overlaps with its DNA-binding surface whereas in RhsP2 it lies adjacent to the RNA-binding surface. This arrangement aligns with the distinct chemical nature of their substrates since DarT modifies the nucleobases of DNA, whereas RhsP2 targets the solvent-exposed 2′-hydroxyl group unique to RNA. As a result, even if RhsP2 binds DNA *via* electrostatic interactions, the absence of this reactive group would preclude ADP-ribosylation, reinforcing its substrate specificity at the level of chemical reactivity. Overall, our findings support a model in which ARTs can evolve diverse nucleic acid targeting capabilities through subtle modifications to their catalytic fold.

Although RhsP2 exhibits broad activity toward structured RNAs, including tRNAs and the RNA subunit of RNase P, the breadth of its physiological targets and substrate selectivity have remained unexplored. Here, we demonstrate that RhsP2 binds and modifies two sRNAs, CsrB and McaS, with distinct affinities. Both sRNAs are highly structured and are functionally important regulators of bacterial gene expression (43, 44). CsrB modulates mRNA stability and translation by antagonizing CsrA, a protein that plays a central role in coordinating the post-transcriptional regulation of bacterial metabolism, motility, and biofilm formation (45, 46, 47, 48, 49). McaS controls biofilm formation and motility through interactions with the Hfq RNA chaperone and other RNA-binding proteins (40, 41, 50). ADP-ribosylation of these RNAs could plausibly interfere with their stability, processing, or protein interactions, thereby contributing to the observed toxicity of RhsP2. Future studies aimed at mapping modification sites and characterizing the functional consequences of ADP-ribosylation will be critical to define the full scope of RhsP2-mediated toxicity.

Interestingly, RhsP2 binds CsrB with tenfold greater affinity than McaS, suggesting that its RNA-binding surface can discriminate between substrates based on features beyond electrostatic complementarity. Given the structural diversity of bacterial RNAs, it is likely that RhsP2 exhibits moderate substrate promiscuity, with preferences shaped by RNA secondary structure, charge distribution, and potential sequence motifs. Substrate-specific effects observed among RNA-binding surface mutants further support a model in which individual basic residues contribute to distinct aspects of RNA recognition. Understanding the structural principles that govern these interactions may offer broader insight into how RhsP2 achieves its modest substrate selectivity and is the focus of ongoing efforts in our lab.

In summary, this work identifies the key molecular features that enable RhsP2 to recognize and modify structured RNAs. These findings provide a mechanistic foundation for understanding RNA-targeting ARTs and establish RhsP2 as a model for exploring non-canonical RNA recognition. In doing so, we expand the known strategies by which bacteria manipulate RNA to gain a competitive advantage.

## Experimental procedures

### Bacterial strains and growth conditions

*P. aeruginosa* strains used in this study were derived from the sequenced strain PA14 (51). Cultures were grown in lysogeny broth (LB) medium (10 g/L tryptone, 10 g/L NaCl, and 5 g/L yeast extract) at 37 °C. Solid LB media contained either 1.5% or 3% (w/v) agar. Media was supplemented with 30 μg/ml gentamicin as needed. *E. coli* strain XL-Blue (Novagen) was used for plasmid maintenance, competition assays, and toxicity experiments. *E. coli* BL21 (pLysS) was used for protein expression and purification. Finally, *E. coli* SM10 was use for conjugative transfer. All *E. coli* strains were grown in LB broth with shaking at 220 RPM or LB agar at 37 °C or 18 °C, as required. Where required, media were supplemented with antibiotics or inducers: 150 μg/ml carbenicillin, 200 μg/ml trimethoprim, 15 μg/ml gentamicin, 100 μg/ml ampicillin, 1.0 mM β-D-1-thiogalactopyranoside (IPTG), or 0.2% (w/v) L-rhamnose. A detailed list of strains used in this study can be found in Table S1.

### DNA manipulation, plasmid construction, and mutant strain generation

All primers were synthesized and purified by Integrated DNA Technologies (IDT). DNA polymerase and restriction enzymes were obtained from New England Biolabs (NEB). Sanger sequencing was performed by TCAG (The Centre for Applied Genomics). Plasmids for heterologous expression included pETDuet-1, pSCrhaB2-CV, and pPSV39-CV and were constructed using standard restriction enzyme-based cloning techniques. A detailed list of plasmids used in this study can be found in Tables S2.

Chromosomal mutants in *P. aeruginosa* were generated by two-step allelic exchange as previously described (52). Briefly, approximately 600 bp flanking sequences upstream and downstream of the mutation site were amplified by PCR and ligated into the allelic exchange vector pEXG2 using Gibson assembly. The construct was transformed into SM10 and subsequently introduced into *P. aeruginosa via* conjugative transfer. The merodiploids were selected on LB agar that contained 5% (w/v) sucrose for the purpose of *sacB* counterselection. Mutants were identified by colony PCR in strains that grew on sucrose but were sensitive to gentamicin. Chromosomal mutations were verified by PCR amplification and Sanger sequencing of the target regions.

### Bacterial toxicity assays

Gene fragments encoding RhsP2_tox_ and its point mutants were ligated into the rhamnose-inducible vector pSCrhaB2-CV and transformed into an *E.coli* XL-blue expressing the immunity protein Rhsl2 from plasmid pPSV39-CV. These cultures were grown to stationary phase overnight at 37 °C in LB media supplemented with 200 μg/ml trimethoprim and 15 μg/ml gentamicin. Cultures were then pelleted, washed with LB, resuspended to an OD_600_ of 0.1, and then serially diluted 10-fold up to 10^-5^. Dilutions were spotted onto LB agar plates containing 250 μM IPTG, 200 μg/ml trimethoprim, and 15 μg/ml gentamicin with or without 0.2% (w/v) L-rhamnose and incubated overnight at 37 °C.

### Competition assays

Interspecific competition assays were performed between *E. coli* (recipient) and *P. aeruginosa* (donor). Stationary phase cultures grown overnight were harvested by centrifugation, washed three times in 2 ml of fresh LB broth, and normalized to an OD_600_ of 1.0. Donor and recipient cultures were then mixed at a 5:1 ratio (v:v), and 10 μl of the mixture was spotted in triplicate onto a 0.45 μm nitrocellulose membrane overlaid on a 3% (w/v) LB agar plate. Plates were incubated face up at 25 °C for 10 h. The competitions were then harvested by scraping each spot from the nitrocellulose and resuspending the cells in LB. Colony-forming units (CFUs) of the surviving *E. coli* were quantified by serially diluting the resuspensions in 10-fold in increments to 10^−6^, followed by plating each dilution onto LB agar plates containing 15 μg/ml gentamicin.

### Electrophoretic mobility shift assay (EMSA)

The 50 nt RNA was synthesized by IDT, and McaS and CrsB were transcribed *in vitro* using the TranscriptAid T7 High Yield Transcription kit (Thermo Scientific) according to the manufacturer’s instructions. For EMSAs, 0.2 μM RNA was incubated with the indicated amounts of wild-type or mutant RhsP2_tox_ protein for 20 min at room temperature in a 10-μl reaction containing 50 mM Tris-HCl pH 7.5, 50 mM NaCl, 1 mM DTT, 5 mM MgCl2, and 4% glycerol. The RNA-protein complexes were separated by 6% (for McaS) or 4% (for CsrB) native PAGE gel at 200 V for 30 min in 0.5× TBE buffer. Gels were stained with SYBR Gold and imaged using a ChemiDoc MP Imaging System (Bio-Rad). Each experiment was performed with at least two independent biological replicates (n ≥ 2). Apparent dissociation constants (K_d,app_) were determined by first calculating band densitometry using ImageJ. Bands representing protein-bound or free RNA were quantified and background-subtracted signals were used to calculate the fraction bound using the following equation: bound/(bound + unbound). These values were plotted against the concentration of wild-type or mutant RhsP2_tox_ and fit by nonlinear regression using Prism 9.0 to obtain K_d,app_ and B_max_ (fraction bound at which the data plateaus) values. The fitting equation used was:

$$\text{Fraction}\;\text{bound}\;\left(\%\right)=\left(\frac{B_{\max}\left[{\text{RhsP}2}_{\text{tox}}\right]}{K_{d}+\left[{\text{RhsP}2}_{\text{tox}}\right]}\right)100$$

### *In vitro* ADP-ribosylation assay and detection of modified RNA

*In vitro* ADP-ribosylation reactions were performed using 50 nt synthetic RNA (Integrated DNA technologies) or *in vitro* transcribed full-length McaS and CsrB. Proteins for the assay were purified as described above. Reactions (10 μl) were assembled in ADP-ribosylation buffer (50 mM Tris-HCl pH 7.5, 50 mM NaCl, 1 mM DTT, and 5 mM MgCl_2_) and contained 0.6 μM protein, 0.2 μM RNA, and 1 mM NAD^+^ or biotin-NAD^+^. Prior to use, RNA samples were stored in nuclease-free ddH_2_O and were refolded immediately before the reaction by heating at 95 °C for 5 min followed by snap cooling on ice for at least 5 min. Reactions were incubated at room temperature for 30 min and stopped by heating at 95 °C for 5 min. Subsequently, the RNA was purified by the RNAClean XP (Beckman Coulter) kit according to the manufacturer’s instructions.

For SYBR Gold staining, 10 μl of 2 μM purified RNA was loaded onto 8% Urea-PAGE gel and run in 0.5× TBE buffer for 30 min at 200V. The gel was then stained with SYBR Gold Nucleic Acid Gel Stain and visualized using a ChemiDoc imaging system. For detection of biotinylated RNA, samples were separated by 8% Urea-PAGE gel and transferred onto Zeta-probe quaternary amine nylon membranes (Bio-Rad) at 100V for 30 min in 0.5× TBE buffer. RNA was crosslinked to the membrane by UV exposure (120 mJ/cm^2^), followed by washing with PBS-T (PBS, 0.05% Tween-2 w/v) with gentle shaking at room temperature, and then incubated in blocking buffer (PBS, 0.05% Tween-20 w/v, 0.2% I-block reagent w/v) for 15 min. Membranes were then incubated with stabilized streptavidin-HRP conjugate (1:300 dilution in 16 ml of blocking buffer) for 15 min with gentle shaking. The membrane was subsequently washed four times with PSS-T (5 min each), followed by development using Luminol/Enhancer solution (Thermo Fisher) for 5 min without shaking. Membranes were visualized using a ChemiDoc instrument (Bio-Rad).

### Northern blotting

Total RNA was extracted using the Quick-RNA Bacterial Microprep Kit (Zymo Research) according to the manufacturer’s instructions. The RNA samples (2 μg) were denatured in RNA loading buffer at 95 °C for 2 min, then loaded onto an 8% urea-PAGE. After separation, the RNA was transferred onto Zeta-probe quaternary amine nylon membranes (Bio-Rad) using the Mini Trans-Blot electrophoretic transfer system (Bio-Rad). RNA was crosslinked to the membrane by UV light exposure at 120 mJ/cm^2^ and immediately incubated in 10 ml pre-heated ULTRAhyb-Oligo (Thermo Fisher) hybridization buffer to 42 °C in a shaking incubator. The membrane was pre-hybridized for 45 min with gentle rotation, followed by the addition of 10 μM biotinylated DNA probe. Hybridization was carried out for 12 h at 42 °C. The membrane was washed twice at 42 °C with stringency wash buffer (2× SSC, 0.5% SDS w/v) under gentle rotation, followed by two additional washes at room temperature with the same buffer. The membrane was then moved to room temperature and washed twice with stringency wash buffer followed by three washes in blocking buffer (2 × SSC, 0.5% SDS w/v, 0.1% I-block reagent w/v). Streptavidin-horseradish peroxidase (HRP) conjugate was diluted 1:5000 in fresh blocking buffer and added to the membrane, followed by a 1-h incubation at room temperature with gentle rotation. The membrane was then washed twice with blocking buffer, followed by three washes with wash buffer. The blot was developed by the addition of Clarity Max ECL substrate (Bio-Rad) and visualized using a ChemiDoc instrument (Bio-Rad).

### RNA pull-down

For the RNA pull-down experiments, RNA was extracted from induced and non-induced *E. coli* cells 30 min after induction using the Quick-RNA Bacterial Microprep Kit (Zymo Research), according to manufacturer’s instructions. RNA was resuspended in 50 μl of 2× hybridization buffer (2× PBS, pH 7.4, 1% SDS w/v, 2 mM EDTA) and subsequently diluted 1:1 with 10 μM 5′-biotinylated DNA probe. Probes were then hybridized to McaS by heating the sample to 95 °C for 5 min using a thermocycler, and then slowly cooling this reaction to 25 °C in 5-degree increments, with a 10-min incubation at each step. Then the hybridization reactions were mixed with 50 μl of pre-washed streptavidin-agarose beads (Sigma) in 1× hybridization buffer. After an overnight incubation at room temperature with gentle shaking, the beads were collected by spinning at 16,000 rpm followed by five washes with stringency wash buffer. Sample were then washed twice with DNase I buffer, and DNA was digested with 4 units DNase at room temperature for 30 min. RNA samples were then purified using Monarch RNA clean-up kit.

### Anti-ADPr immunoblotting

Following pull-down of RNA from *E. coli* expressing empty-vector or RhsP2_tox_, the RNA was spotted onto a dry Zeta-Probe quaternary amine nylon membrane and cross-linked by UV light exposure at 120 mJ/cm^2^. Membranes were washed with PBS-T (PBS, 0.05% Tween-2 w/v) by shaking gently at room temperature and then incubated in blocking buffer (PBS, 0.05% Tween-20 w/v, 0.2% I-block reagent w/v) for 1 h. The membrane was then placed in fresh blocking buffer supplemented with monoclonal poly/mono-ADPr rabbit antibody (diluted 1:2500) (Cell Signaling Technology) and incubated overnight at 4 °C with gentle shaking. Following incubation, the membrane was washed with PBS-T three times for five-minute increments. Following washing, secondary antibody (anti-rabbit HRP conjugate) was added in a 1:5000 dilution to fresh PBS-T and incubated at room temperature for an hour with gentle shaking. The membrane was then washed with PBS-T thrice, developed using Clarity Max ECL substrate (Bio-Rad) and visualized using a ChemiDoc instrument (Bio-Rad).

### Quantitative real-time RT‒PCR (qRT‒PCR) analysis

To determine the expression of the McaS in toxin inducted and control conditions, total RNA was isolated as described above. Subsequently, total RNA was reverse transcribed using random primers and the High-Capacity cDNA Reverse Transcription Kit (Thermo Fisher Scientific) according to the manufacturer’s instructions. qPCR was carried out with Power SYBR Green PCR Master Mix on Real-Time PCR system (Bio-Rad), following the manufacturer’s recommendations. The 16S rRNA gene was used as the endogenous reference control, and relative gene expression levels was calculated using the comparative threshold cycle 2^−ΔΔCT^ method. The primers for qPCR are listed in Table S3.

### Protein expression and purification

For purification of wild-type RhsP2_tox_ and its point mutants, all variants of the effector were co-expressed alongside the RhsP2 immunity protein on pETDuet, to prevent cellular intoxication. An on-column denaturation and refolding strategy was then used to remove the immunity protein, allowing for the purification of the N-terminal His-tagged effector. For each toxin-immunity pair, 1 L of LB medium containing 100 μg/ml ampicillin was inoculated with *E. coli* BL21 pLysS cells harboring the toxin-immunity pETDuet construct. Cultures were grown shaking at 37 °C with shaking until reaching an OD_600_ of 0.5 to 0.8. The temperature was then reduced to 18 °C, and IPTG was added to a final concentration of 1 mM to induce expression. After overnight incubation, cells were harvested by centrifugation at 4500 RPM for 15 min and resuspended in lysis buffer (50 mM Tris-HCl, pH8.0, 300 mM NaCl, 10 mM imidazole). Cells were lysed by sonication and the lysates were clarified by centrifugation at 15,000 RPM for 45 min. The supernatant was loaded onto a 1.5 ml gravity flow Ni-NTA agarose column pre-equilibrated with lysis buffer. The column bound effector-immunity complex was denatured with 8 M urea supplemented in the lysis buffer, facilitating removal of the untagged immunity protein. Following denaturation, the effector was renatured by washing the column three times lysis buffer. The refolded protein was eluted using 10 ml of lysis buffer supplemented with 400 mM imidazole. The purity of each protein was determined by SDS-PAGE gel followed by staining with Coomassie Brilliant Blue. Imidizole was removed from the final storage buffer (20 mM Tris pH 7.5 and 150 mM NaCl) through dialysis.

### Isothermal titration calorimetry

Binding experiments were performed using a MicroCal PEAQ-ITC microcalorimeter (Malvern). Prior to the experiment, proteins were dialyzed overnight at 4 °C against 50 mM HEPES pH 7.4, 300 mM NaCl, 5% glycerol using dialysis. NAD^+^ was prepared in the same buffer used for protein dialysis. Titration experiments were conducted at 25 °C with a reference power of 12 μCal/s and a stirring speed of 750 rpm. The protocol consisted of an initial injection of 0.4 μl, followed by 18 identical injections of 4 μl each (duration of 4 s per injection and spacing of 240 s between injections). Data were analyzed using the MicroCal PEAQ-ITC analysis software (Malvern).

### Quantification and statistical analysis

Statistical analysis of bacterial competitions was performed using GraphPad Prism 9.0. One-way ANOVA was used for significance testing. A *p*-value of *p* ≤ 0.05 was considered statistically significant. Data are presented as averages ± SEM. Significance levels are indicated as follows: ∗∗∗∗*p* ≤ 0.0001; ∗∗∗*p* ≤ 0.001; ∗∗*p* ≤ 0.01; ∗*p* ≤ 0.05. Each bar in graphs represents the mean of biological replicates and error bars indicate the SEM (standard error of the mean). For all assays, *n* represents the number of biological replicates.

## Data availability

All data supporting the findings of this study are available within the manuscript and its associated supplementary information.

## Supporting information

This article contains supporting information (53, 54, 55).

## Conflict of interest

The authors declare that they have no conflicts of interest with the contents of this article.
