# Supplementary material for: Molecular determinants of nucleic acid recognition by an RNA-targeting ADP-ribosyltransferase toxin
Source: J Biol Chem. 2025 Jul 7;301(8):110463. doi: 10.1016/j.jbc.2025.110463 (PMC12340436; doi:10.1016/j.jbc.2025.110463)
Supplement: Supplemental information [file mmc1.pdf]

Figure S1. A) Predicted Aligned Error (PAE) plots generated by AlphaFold3 for the indicated RhsP2-NAD^+ complexes. Color corresponds to expected position error as indicated by the scale bar. **B)** Structural alignment of $RhsP2_{tox}$ (green) with the NAD^+ binding and catalytic domain of diphtheria toxin (yellow). Conserved motif residues and additional active site residues essential for catalysis are shown in green, along with their relative positioning to the NAD^+ substrate. **C)** Isothermal titration calorimetry (ITC) measurements of NAD^+ binding affinity for NAD^+ was titrated into the respective protein in ITC buffer. The top panel shows the raw titration data, and the bottom panel represents the fitted Wiseman isotherm of integrated peaks.

Figure S2. Electrophoretic mobility shift assays (EMSAs) analyzing the RNA-binding activity of $RhsP2_{tox}$ mutants. The raw gel images showing the binding of a 50-nt RNA to various $RhsP2_{tox}$ mutants, separated on an 6% native PAGE gel and visualized using SYBR Gold staining.

Figure S3. A) Sequence alignment of McaS and CsrB RNAs.
(B–D) Predicted secondary structures of CsrB (B), synthetic 50-nt RNA (C), and McaS (D), generated using RNAfold.

Figure S4. A and B) EMSAs assessing the RNA-binding activity of $RhsP2_{tox}$ mutants using full-length McaS (A) and CrsB (B) as substrates. RNA-protein complexes were resolved on native PAGE gels (6% for McaS and 4% for CsrB) and visualized by SYBR Gold staining.

Table S1: Strains used in this study

Organism	Genotype	Description	References
Pseudomonas aeruginosa PA14	Wild type		(30)
	ΔPA14_52570 ΔPA14_20290	Δ*rsmA*Δ*amrZ* deletion strain	(30)
	ΔPA14_52570 ΔPA14_20290 ΔPA14_43100	Δ*rsmA*Δ*amrZ*Δ*rhsP2* deletion strain	(30)
	ΔPA14_52570 ΔPA14_20290	Δ*rsmA*Δ*amrZ* deletion, *rhsP2*^{R1563E} strain	This study
	ΔPA14_52570 ΔPA14_20290	Δ*rsmA*Δ*amrZ* deletion, *rhsP2*^{K1564E} strain	This study
	ΔPA14_52570 ΔPA14_20290	Δ*rsmA*Δ*amrZ* deletion, *rhsP2*^{K1567E} strain	This study
	ΔPA14_52570 ΔPA14_20290	Δ*rsmA*Δ*amrZ* deletion, *rhsP2*^{K1574E} strain	This study
	ΔPA14_52570 ΔPA14_20290	Δ*rsmA*Δ*amrZ* deletion, *rhsP2*^{K1579E} strain	This study
	ΔPA14_52570 ΔPA14_20290	Δ*rsmA*Δ*amrZ* deletion, *rhsP*2^{K1580E} strain	This study
E. coli BL21 (DE3) pLysS	F- ompT gal dcm lon hsdSB(rB$^-$ mB$^-$) λ (DE3) pLysS(cm^R)	Protein expression strain	Novagen
E. coli SM 10λpir	*thi thr leu tonA lac Y supE recA*::RP4-2-Tc::Mu	Conjugation strain	BioMedal LifeScience
E. coli XL-1 Blue	*recA1 endA1 gyrA96 thi-1 hsdR17 supE44 relA1 lac* [F′ *proAB lacI*^{q}Z*ΔM15* Tn*10* (Tet^R)]	Cloning strain	Novagen

Table S2: Plasmids used in this study

Plasmid	Relevant features	Reference
pEXG2	Allelic replacement vector containing *sacB*, Gm^R	(53)
pEXG2::RhsP2_R1563E	*rhsP2* R1563E point mutant construct	This study
pEXG2::RhsP2_K1564E	*rhsP2* K1564E point mutant construct	This study
pEXG2::RhsP2_K1567E	*rhsP2* K1567E point mutant construct	This study
pEXG2::RhsP2_K1574E	*rhsP2* K1574E point mutant construct	This study
pEXG2::RhsP2_K1579E	*rhsP2* K1579E point mutant construct	This study
pEXG2::RhsP2_K1580E	*rhsP2* K1580E point mutant construct	This study
pSCrhaB2-CV	Expression vector with *PrhaB*, Tmp^R	(55)
pSCrhaB2-CV::PA14_43100_1445-CT	Expression vector for $RhsP2_{tox}$	This study
pSCrhaB2-CV::PA14_43100_1445-CT_E1576Q	Expression vector for $RhsP2_{tox}^{E1576Q}$	(30)
pSCrhaB2-CV::PA14_43100_1445-CT_R1490A	Expression vector for $RhsP2_{tox}^{R1490A}$	(30)
pSCrhaB2-CV::PA14_43100_1445-CT_Y1524A	Expression vector for $RhsP2_{tox}^{Y1524A}$	(30)
pSCrhaB2-CV::PA14_43100_1445-CT_R1563E	Expression vector for $RhsP2_{tox}^{R1563E}$	This study
pSCrhaB2-CV::PA14_43100_1445-CT_K1564E	Expression vector for $RhsP2_{tox}^{K1564E}$	This study
pSCrhaB2-CV::PA14_43100_1445-CT_K1567E	Expression vector for $RhsP2_{tox}^{K1567E}$	This study
pSCrhaB2-CV::PA14_43100_1445-CT_K1574E	Expression vector for $RhsP2_{tox}^{K1574E}$	This study
pSCrhaB2-CV::PA14_43100_1445-CT_K1579E	Expression vector for $RhsP2_{tox}^{K1579E}$	This study
pSCrhaB2-CV::PA14_43100_1445-CT_K1580E	Expression vector for $RhsP2_{tox}^{K1580E}$	This study
pPSV39-CV	Expression vector with lacI, lacUV5 promoter, C-terminal VSV-G tag, GmR	(54)
pPSV39-CV::PA14_RS30650	Expression vector for *rhsI2*	(30)
pETDuet	Co-expression vector with lacI, T7 promoter, N-terminal His6 tag in MCS-1, Amp^R	Novagen
pETDuet::PA14_43100_1445- CT::PA14_RS30650	Co-expression vector of $RhsP2_{tox}$ and RhsI2	(30)
pETDuet::PA14_43100_1445-CT_R1490A::PA14_RS30650	Co-expression vector of $RhsP2_{tox}^{R1490A}$ and RhsI2	This study
pETDuet::PA14_43100_1445-CT_Y1524A::PA14_RS30650	Co-expression vector of $RhsP2_{tox}^{Y1524A}$ and RhsI2	This study
pETDuet::PA14_43100_1445-CT_R1563E::PA14_RS30650	Co-expression vector of $RhsP2_{tox}^{R1563E}$ and RhsI2	This study
pETDuet::PA14_43100_1445-CT_K1564E::PA14_RS30650	Co-expression vector of $RhsP2_{tox}^{K1564E}$ and RhsI2	This study
pETDuet::PA14_43100_1445-CT_K1567E::PA14_RS30650	Co-expression vector of $RhsP2_{tox}^{K1567E}$ and RhsI2	This study
pETDuet::PA14_43100_1445-CT_K1574E::PA14_RS30650	Co-expression vector of $RhsP2_{tox}^{K1574E}$ and RhsI2	This study
pETDuet::PA14_43100_1445-CT_K1579E::PA14_RS30650	Co-expression vector of $RhsP2_{tox}^{K1579E}$ and RhsI2	This study
pETDuet::PA14_43100_1445-CT_K1580E::PA14_RS30650	Co-expression vector of $RhsP2_{tox}^{K1580E}$ and RhsI2	This study

Table S3: Primers used in this study

Primer name	Sequence (5’ to 3’)	Description
RT-McsA F	CGGATTTAAGACGCGGATGC	qRT-PCR for McsA
RT-McsA R	CGACATCCGCCAGACTCTAC	
16S F	TACCGCATAACGTCGCAAGA	qRT-PCR for 16S rRNA
16S R	CTGGACCGTGTCTCAGTTCC	
T7-McaS F	TAATACGACTCACTATAGGGACCGGCGCAGAGGAG	In vitro transcription for McaS
T7-McaS R	AAAAAAATAGAGTCTGTCGACATCCGCCAGACTCTACAGT	
T7-CsrB F	TAATACGACTCACTATAGGGgtcGACAGGGAGTCAG	In vitro transcription for CsrB
T7-CsrB R	aatAAAAAAAGGGAGCACTGTATTCACAGCGCTCCC	
R1563E up F	TTCCACACATTATACGAGCCGGAAGCATAAATGTAAAGCAAGCTTCGACCACCTCGGTACCCC	For construction of pEXG2::RhsP2$_{tox}^{R1563E}$
R1563E up R	ATAGTGCGTTGTTCTGTTTCCAGCCCTTTTCGGCGGG	
R1563E down F	CCTCCCGCCGAAAAGGGCTGGAAACAGAACAACGCACTATTCAAACT	
R1563E down R	GCACGATCATGCGCACCCGTGGAAATTAATTAAGGTACCGAATTCCCGATGTATGATTCCCATGG	
R1564E up F	CCGGAAGCATAAATGTAAAGCAAGCTTCGACCACCTCGGTACCCC	For construction of pEXG2::RhsP2$_{tox}^{R1564E}$
R1564E up R	GTTCTGTTTCCAGCCCTCTCGGGCG	
R1564E down F	CCGAGAGGGCTGGAAACAGAACAACGCACTATTCAAACT	
R1564E down R	CCGTGGAAATTAATTAAGGTACCGAATTCCCGATGTATGATTCCCATGGATAGGG	
K1567E up F	TTCCACACATTATACGAGCCGGAAGCATAAATGTAAAGCAAGCTTCGACCACCTCGGTACCCC	For construction of pEXG2::RhsP2$_{tox}^{K1567E}$
K1567E up R	TTTAGTGCTTCGAGTTTGAATAGTGCGTTGTTCTGTTCCCAGCCC	
K1567E down F	CGAAAGGGCTGGGAACAGAACAACGCACTATTCAAACTCGAAGCACTAAAAAAGCCA	
K1567E down R	GCACGATCATGCGCACCCGTGGAAATTAATTAAGGTACCGAATTCCCGATGTATGATTCCCATGG	
K1574E up F	CCGGAAGCATAAATGTAAAGCAAGCTTCGACCACCTCGGTACCCC	For construction of pEXG2::RhsP2$_{tox}^{K1574E}$
K1574E up R	GCTTTTTTAGTGCTTCGAGTTCGAATAGTGCGTTGT	
K1574E down F	ATTCGAACTCGAAGCACTAAAAAAGCCAACGATAAATGAAGGAGG	
K1574E down R	CCGTGGAAATTAATTAAGGTACCGAATTCCCGATGTATGATTCCCATGGATAGGG	
K1579E up F	CCGGAAGCATAAATGTAAAGCAAGCTTCGACCACCTCGGTACCCC	For construction of pEXG2::RhsP2$_{tox}^{K1579E}$
K1579E up R	GCTTTTCTAGTGCTTCGAGTTTGAATAGTGCGTTGTTCT	
K1579E down F	CAAACTCGAAGCACTAGAAAAGCCAACGATAAATGAAGGAG	
K1579E down R	CCGTGGAAATTAATTAAGGTACCGAATTCCCGATGTATGATTCCCATGGATAGGG	
K1580E up F	GGAAGCATAAATGTAAAGCAAGCTTCGACCACCTCGGTACCCC	For construction of pEXG2::RhsP2$_{tox}^{K1580E}$
K1580E up R	GCTCTTTTAGTGCTTCGAGTTTGAATAGTGCGTTGTTCT	
K1580E down F	AAACTCGAAGCACTAAAAGAGCCAACGATAAATGAAGGAG	
K1580E down R	GTGGAAATTAATTAAGGTACCGAATTCCCGATGTATGATTCCCATGG	

	ATAGGG	
pETDuet K1564E up F	catcaccacagccaggatccgCTGGCCTCAAATAAAACTCGCTGT	For construction of pETDuet::RhsP2$_{tox}^{K1564E}$::PA14_RS30650
pETDuet K1564E up R	TGTTTCCAGCCCTCTCGGGCGG	
pETDuet K1564E down F	GAGAGGGCTGGAAACAGAACAACGCACTATTCAAACT	
pETDuet K1564E down R	cattatgcggccgcaagcttCTAGTCTATTACTTCGAAGTCTATTAGGTTCTTGTTGA	
pETDuet K1567E up F	catcaccacagccaggatccgCTGGCCTCAAATAAAACTCGCTGT	For construction of pETDuet::RhsP2$_{tox}^{K1567E}$::PA14_RS30650
pETDuet K1567E up R	TGTTCCCAGCCCTTTCGGGCGGG	
pETDuet K1567E down F	AAAGGGCTGGGAACAGAACAACGCACTATTCA	
pETDuet K1567E down R	cattatgcggccgcaagcttCTAGTCTATTACTTCGAAGTCTATTAGGTTCTTGTTGA	
pETDuet K1574E up F	catcaccacagccaggatccgCTGGCCTCAAATAAAACTCGCTGT	For construction of pETDuet::RhsP2$_{tox}^{K1574E}$::PA14_RS30650
pETDuet K1574E up R	CGAATAGTGCGTTGTTCTGTTTCCAGCCCT	
pETDuet K1574E down F	AGAACAACGCACTATTCGAACTCCAAGCACTAAAAAAGC	
pETDuet K1574E down R	cattatgcggccgcaagcttCTAGTCTATTACTTCGAAGTCTATTAGGTTCTTGTTGA	
pETDuet K1579E up F	catcaccacagccaggatccgCTGGCCTCAAATAAAACTCGCTGT	For construction of pETDuet::RhsP2$_{tox}^{K1579E}$::PA14_RS30650
pETDuet K1579E up R	CTTTTCTAGTGCTTGGAGTTTGAATAGTGCGTTGT	
pETDuet K1579E down F	AACTCCAAGCACTAGAAAAGCCAACGATAAATGAAGGAG	
pETDuet K1579E down R	cattatgcggccgcaagcttCTAGTCTATTACTTCGAAGTCTATTAGGTTCTTGTTGA	
pETDuet K1580E up F	catcaccacagccaggatccgCTGGCCTCAAATAAAACTCGCTGT	For construction of pETDuet::RhsP2$_{tox}^{K1580E}$::PA14_RS30650
pETDuet K1580E up R	GCTCTTTTAGTGCTTGGAGTTTGAATAGTGCGT	
pETDuet K1580E down F	TCCAAGCACTAAAAGAGCCAACGATAAATGAAGGAGGC	
pETDuet K1580E down R	cattatgcggccgcaagcttCTAGTCTATTACTTCGAAGTCTATTAGGTTCTTGTTGA	
pETDuet R1563E up F	ccatcatcaccacagccaggatccCTGGCCTCAAATAAAACTCGCTGT	For construction of pETDuet::RhsP2$_{tox}$

pETDuet R1563E up R	CTGTTTCCAGCCCTTTTCGGCGGGAGG	^{R1563E}::PA14_RS30650
pETDuet R1563E down F	CGAAAAGGGCTGGAAACAGAACAACGCACTATTC	
pETDuet R1563E down R	cattatgcggccgcaagcttCTAGTCTATTACTTCGAAGTCTATTAGGTTCTTGTTGA	
