# Supplementary figures and images for: Molecular determinants of nucleic acid recognition by an RNA-targeting ADP-ribosyltransferase toxin
Source: J Biol Chem. 2025 Jul 7;301(8):110463. doi: 10.1016/j.jbc.2025.110463 (PMC12340436; doi:10.1016/j.jbc.2025.110463)

Figure S1

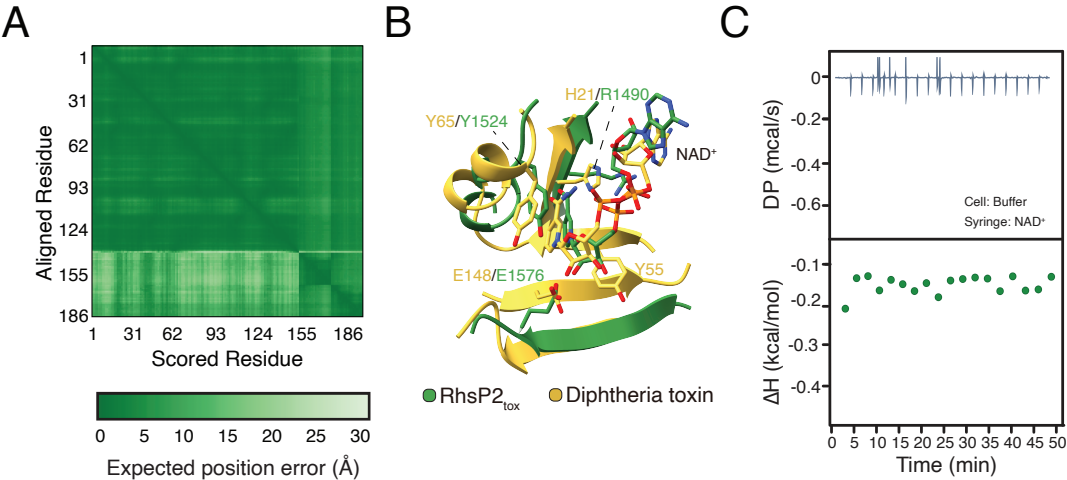

Supplement: Supplemental figure 1 [file mmc2.pdf]

Figure S2

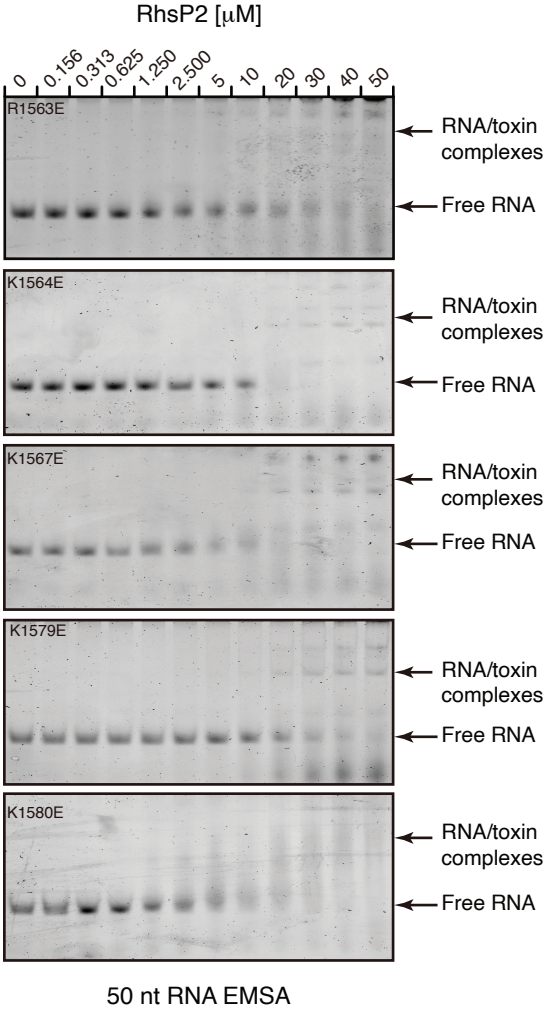

Supplement: Supplemental figure 2 [file mmc3.pdf]

Figure S3

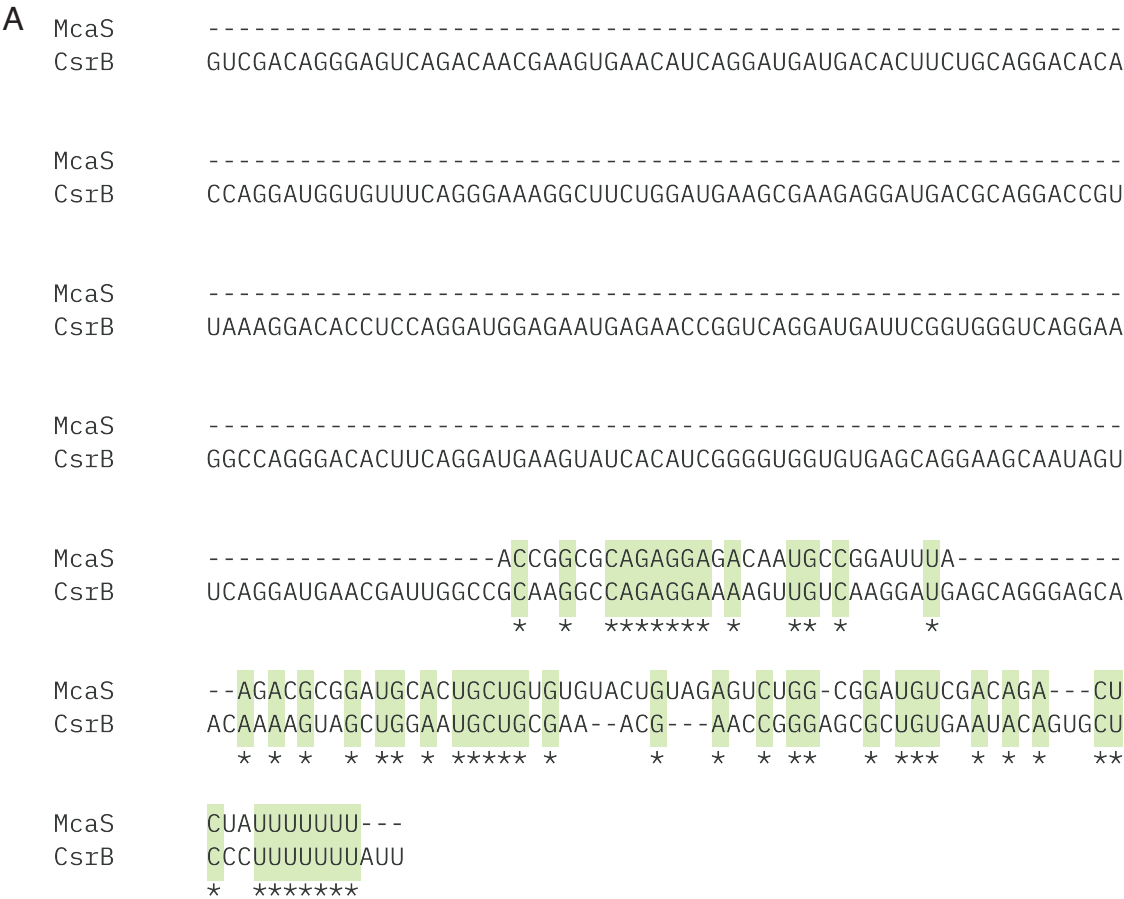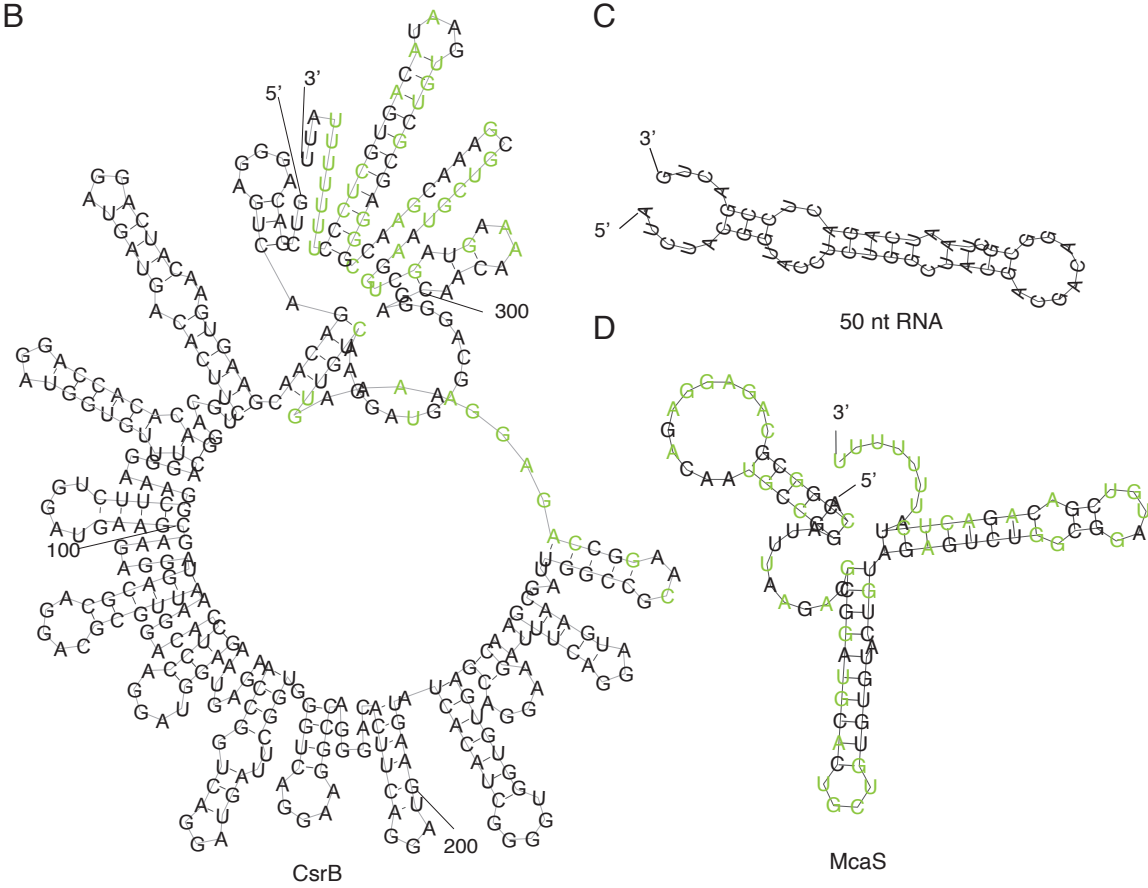

Supplement: Supplemental figure 3 [file mmc4.pdf]

Figure S4

A

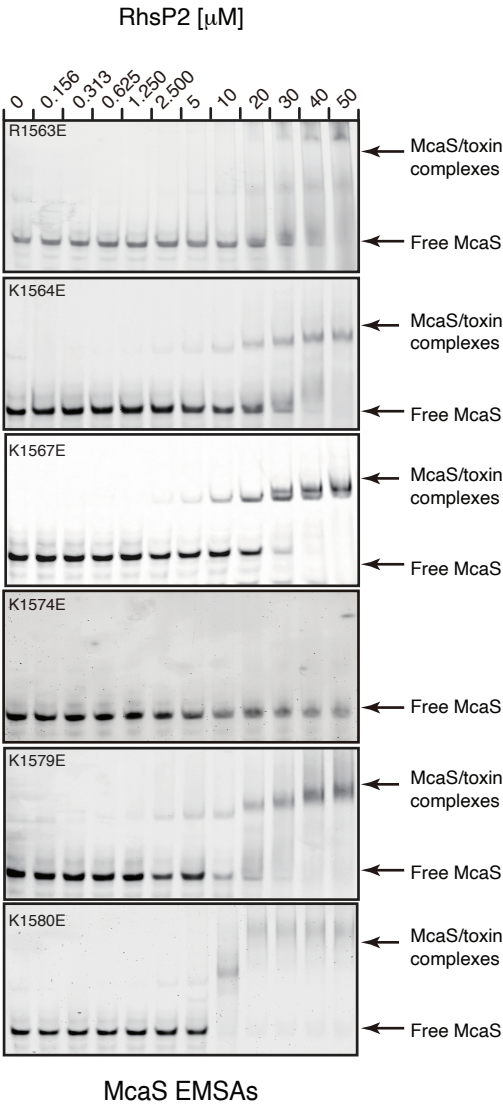

B

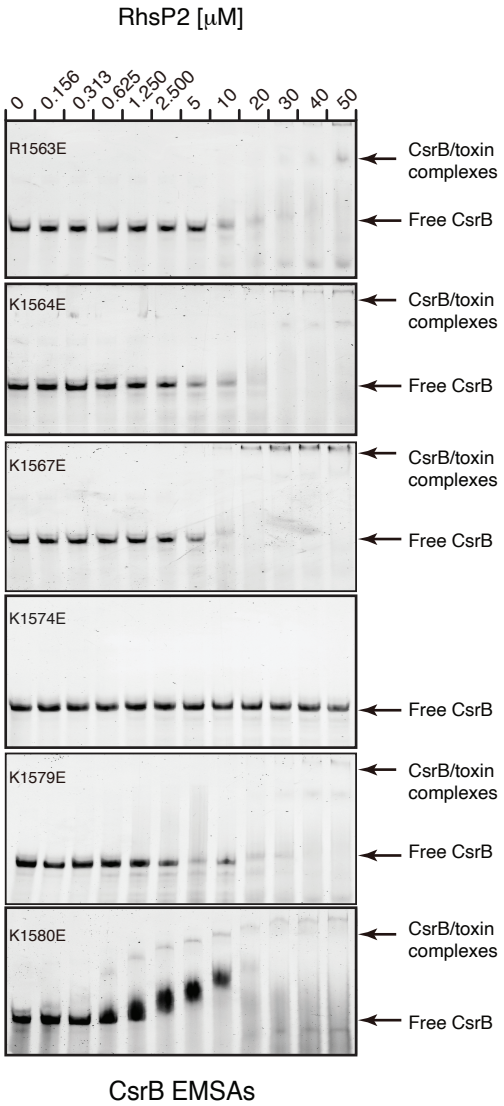

Supplement: Supplemental figure 4 [file mmc5.pdf]
